# Complex Synergistic Interactions among Volatile and Phenolic Compounds Underlie the Effectiveness of Allelopathic Residues Added to the Soil for Weed Control

**DOI:** 10.3390/plants11091114

**Published:** 2022-04-20

**Authors:** María Pardo-Muras, Carolina G. Puig, Nuria Pedrol

**Affiliations:** Department of Plant Biology and Soil Science, Faculty of Biology, University of Vigo, 36310 Vigo, Spain; cgpuig@uvigo.es (C.G.P.); pedrol@uvigo.es (N.P.)

**Keywords:** allelopathy, interaction bioassays, phenolic compounds, soil, VOCs, weed control

## Abstract

The introduction of allelopathic cover crops for green manuring or mulching is a regular practice in Integrated Weed Management. In this context, the alternative use of the abundant phytotoxic residues of allelopathic plants from the agroecosystem, e.g., the foliage of *Eucalyptus*, *Acacia*, or *Cytisus* species, is promising. Previous studies identified the phytotoxic compounds potentially involved in the effectiveness of some plant residues when added to the soil for weed control. The low quantities of allelochemicals present in the tissues and the weak phytotoxicity of each of them in their natural concentrations did not explain the significant levels of weed control observed at field scale. Here, to study hypothetical synergistic interactions among the volatile (VOCs) and water-soluble compounds released to the soil matrix, complex mixtures of VOCs, phenolics, or both, mimicking the chemical profiles of *Cytisus scoparius* were prepared and then tested in vitro on the germination and early growth of two weeds. The effects were calibrated against the VOCs naturally emitted by the fresh plant material and aqueous extract, acting together or not, and with or without soil. The presence of the aqueous extract significantly increased the phytotoxicity of VOCs on *Amaranthus retroflexus* root growth compared to the volatiles emitted alone. In addition, the soil factor enhanced synergistic interactions among VOCs and water-soluble compounds, resulting in a 54% decrease in total germination and an 80% inhibition of root and shoot growth. Multi-level synergistic chemical interactions should explain the bioherbicidal effectiveness of allelopathic residues applied as a soil amendment.

## 1. Introduction

For more than 70 years, weeds have been primarily managed with synthetic herbicides [1]; however, their long-term use with the same mode of action (MOA) has promoted the appearance of herbicide-resistant weeds [2]. Currently, there are more than 500 weed biotypes that have evolved resistance to 21 of the 31 known herbicide sites of action and 164 different herbicides [3]; moreover, no herbicides with real new molecular targets have come on the market in the past 30 years [4,5]. The emergence of herbicide resistance and harmful environmental effects have increased social concern about their use and stimulated interest in discovering new bio-inspired methods to control weeds.

In this context, bioactive plant secondary metabolites could be an alternative to existing synthetic herbicides. Such natural compounds play essential roles in plant defense, including defense against other plants by releasing phytotoxic compounds from plant tissues [6], i.e., allelochemicals that interfere with normal germination and growth [7]. Besides, these compounds present a great structural diversity, with slight MOAs overlapping with synthetic herbicides [8], and are *a priori* environmentally friendly [2]. It is noteworthy that chemical interactions among plants are usually due to complex mixtures of various compounds [9]; therefore, the interactions among allelochemicals have to be explored since small quantities of compounds of different chemical classes and MOAs could be combined to increase herbicide efficiency and minimize the development of resistance.

In Agriculture, Chemistry, and Plant Sciences, studies dealing with interactions among phytotoxic compounds are still very scarce. This deficit could be due to the difficulty of accurately mimicking mixtures of compounds in the lab, besides the lack of well-defined reference models [6,10,11]; moreover, the combined action of natural compounds can be additive, antagonistic, or synergistic, thus adding complexity to the discussion. For example, Rial et al. [12], when assayed seventeen binary combinations of sesquiterpene lactones on etiolated wheat coleoptiles, observed a high level of growth inhibition caused by the mixtures not comparable to that of a single active compound. Otherwise, Inderjit et al. [10] found that the effects of paired combinations of the phenolic acids *p*-hydroxybenzoic, *p*-coumaric, and ferulic on the root growth of *Lolium perenne* depended on the concentration of each compound in the mixture since the compounds might act antagonistically at high concentrations, but additively at low concentrations. Chotsaeng et al. [13] observed synergistic effects on the germination and growth of *Amaranthus tricolor* when compounds of different classes: xanthoxylene, vanillin, limonene, and linoleic acid, were used in combination. More recently, Bravetti et al. [14], after assaying binary and multi-compound mixtures of six phenolic acids, found that ferulic/*p*-coumaric acid in molar ratio 5:2 was the most potent inhibitor of *Solanum lycopersicum* germination, suggesting that the synergistic effect depended on the acids and their relative proportion. On the other hand, studies combining the essential oil or volatile extract and the water-soluble fraction of the same species are practically unheard of. As far as we know, only Dias and Moreira [15] tested the combined activity of the aqueous extracts and some volatile organic compounds (VOCs) emitted by *Cistus ladanifer* leaves. 

The role of soil in the bioactivity of the allelochemicals once released into the environment is critical since many compounds that are effective in vitro could have little or no effectiveness in the field, due to different abiotic and biotic processes [6,16]. Although efforts have been made to determine the permanence of water-soluble and volatile phytochemicals released into soil water and pores [14,17,18], no thorough study has been performed on their interactions, which may result from two or more natural compounds combined into the soil matrix. 

In the search for new effective strategies for weed control, the use of foliage from allelopathic species present in the agroecosystem and capable of releasing natural cocktails of allelochemicals is very promising [19,20,21]. During the decomposition of plant tissues into the agricultural soil, several different compounds, volatile or water-soluble, would potentially act together and sequentially throughout the time [18] and would then provide many different MOAs against the diversity of weeds in the soil seed bank. For instance, the leguminous shrub species *Cytisus scoparius* (L.) Link. (Scotch broom), native to the Atlantic region and highly invasive in other biogeographical regions has demonstrated to be a source of very diverse natural compounds with relevant phytotoxic activity, showing different target physiological processes that were specific in some cases [22,23]. The concentration of each allelochemical quantified in the volatile [22], or aqueous extracts [23] was much lower than the minimum inhibitory concentration of any compound tested alone, suggesting the phytotoxicity of *C. scoparius* was due to the joint action of its allelochemicals rather than to individual activities. Pardo-Muras et al. [24] observed powerful paired synergistic interactions between some of the VOCs that notably increased their individual phytotoxicity on the target weeds *Amaranthus retroflexus* L. and *Digitaria sanguinalis* (L.) Scop.; however, knowing the small quantities of each allelochemical in the plant tissues [22,23], the available results are not enough to explain the significant weed control of *C. scoparius* flowering foliage when used as a soil amendment in pot experiments [25]. These findings and unresolved issues have led us to hypothesise that the herbicidal effectiveness of *C. scoparius* residues and those of other allelopathic species might be due not only to interactions among their different volatiles, on the one hand, or water-soluble compounds, on the other hand, but also to interactions between both families of compounds. Indeed, in Nature, the phytotoxicity observed in many physiological processes was argued to result from the combination and interaction of different allelochemicals [6,26,27], although such argumentation still required empirical support. Thus, it was time to address the multi-level interactions in natural compounds’ richness and to investigate their joint bioactivity.

Therefore, the objectives of the present study were: (i) to test the phytotoxicity of complex mixtures of VOCs, on the one hand, and phenolic compounds, on the other hand, prepared in the lab by mimicking the known volatile and phenolic profiles of *C. scoparius*; (ii) to elucidate in vitro if the volatile and the phenolic fractions interacted when applied concomitantly; (iii) to calibrate all the responses against the volatile emission of fresh plant material and its aqueous extract; and (iv) to emulate the effect of soil on the interactions that might occur.

## 2. Results and Discussion

### 2.1. Phytotoxicity of the Mimicked Volatile Fraction

In the present work, we aimed to shed light on those phytotoxic effects that were hypothesised to derive from interactions among the bioactive compounds of different classes identified from *C. scoparius*. For this purpose, we prepared the mimicked volatile mixtures present in *C. scoparius* flowering branches by maintaining the natural proportions in the chemical profile. It should be noted that An et al. [28] found that a given mixture of allelochemicals in equal proportions was less inhibitory than the combination of the same allelochemicals at their natural proportions, even when the proportion of certain highly phytotoxic compounds was much lower in the natural mixture; therefore, the contribution of each compound to the whole matrix would be affected not only by its concentration and phytotoxic strength but most likely by the mixture’s natural composition. 

**Figure 1:**
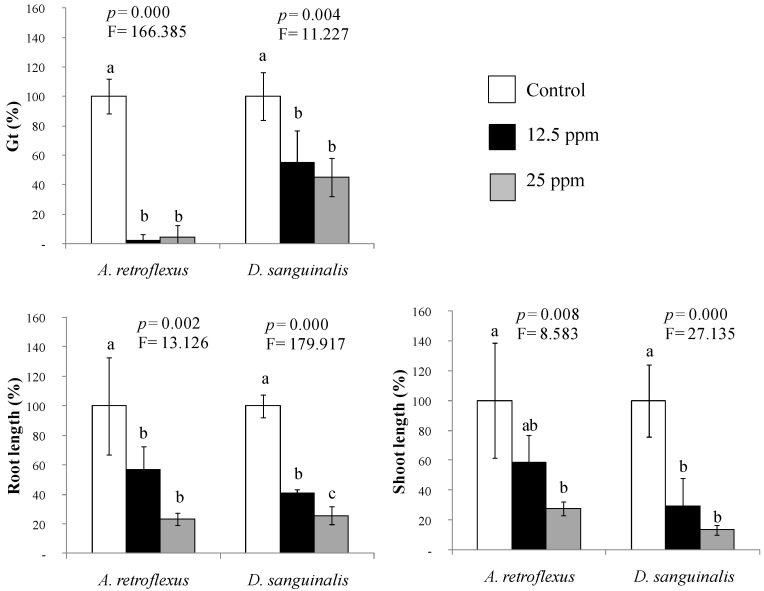
Effects of a mixture of VOCs, prepared by mimicking the volatile profile of *Cytisus scoparius* flowering foliage or only flowers, assayed at 12.5 and 25 ppm in Petri dishes, on the total percentage of germinated seeds (Gt) and early growth of two agricultural weed species (*Amaranthus retroflexus* and *Digitaria sanguinalis*). Mean values of four replicates are represented in percentage relative to control. Error bars represent standard deviation (S.D.). For each target weed species, *p*- and F-values of the effects of treatments from one-way ANOVA or Kruskal-Wallis *H* tests are shown. Mean values labeled with distinct letters denote statistically significant differences among treatments (post hoc LSD or Tamhane’s *T*2 test).

Here, the *C. scoparius* VOCs mixture, consisting of verbenone: *α*-terpineol: linalool: terpinen-4-ol (23:34:29:14), significantly inhibited the germination and early growth of both agricultural weeds (Figure 1). The joint action of 12.5 ppm of the mixture (1 µL added to the filter paper strip fixed inside the top lid of the plate) was enough to prevent the germination of *A. retroflexus* and to reduce that of *D. sanguinalis* by 50% with a significant germination delay (Table 1). Reductions of root and shoot elongation above 60% were highly significant for *D. sanguinalis* (*p* ≤ 0.001) but still very significant for *A. retroflexus* (*p* ≤ 0.01), achieving inhibitions from 43–77% of control (Figure 1).

In the case of *D. sanguinalis*, the VOCs mixture resulted in more phytotoxic than expected from the known paired interactions of its compounds [24]. In comparison, the trend of the inhibitory effects was consistent with previous studies on the VOCs naturally emitted by the fresh plant material in hermetic chambers [22]. Otherwise, the mimicked mixture resulted in highly phytotoxic for *A. retroflexus* if compared to previous data, here preventing germination almost totally. Having in mind the IC_80_ values of the isolated compounds from the dose-response curves obtained in Pardo-Muras et al. [22] for *A. retroflexus* germination (7.4, 18.9, 22.4, and 37.65 ppm for verbenone, *α*-terpineol, linalool, and terpinen-4-ol, respectively), the effects of the mimetic mixture observed here must have involved significant synergistic interactions among its constituents in a restricted atmosphere. Notice that their concentrations in the Petri dish inoculated with 1 µL were much lower (2.9, 4.25, 3.6, and 1.75 ppm for verbenone, *α*-terpineol, linalool, and terpinen-4-ol, respectively). Similar observations apply to the notable inhibitory effects observed here for root and shoot lengths. In the same direction, Araniti et al. [29] observed that the addition of the terpenoids pulegone or farnesene to a mixture of camphor + trans-caryophyllene caused a synergistic action on the root growth of *Arabidopsis thaliana*, the mixtures being in natural proportions according to *Calamintha nepeta* methanolic extract and essential oil. Recently, Koiou et al. [30] observed synergistic effects on germination and root length of *Setaria verticillata* when carvacrol or eugenol was combined with ocimene, 3-octanone, *α*-terpineol, or terpinen-4-ol. 

At this point, it is worth emphasizing the difficulty of reproducing the combined action of allelochemicals in Nature [6,10,11]. In addition, several compounds previously found in the volatile extracts of *C. scoparius* flowering foliage were not included in the mimicked VOCs mixture here due to their reported scarce or null individual phytotoxicity (e.g., some aliphatic compounds, monoterpene hydrocarbons, or other oxygenated mono and diterpenes; [22]); thus, we do not discard the loss of possible complex antagonistic interactions among various VOCs not included in the mimicked *C. scoparius* VOCs mixture. Otherwise, current experimental approaches with pure compounds added in Petri dishes or hermetic chambers imply the immediate release of the VOCs into a controlled and confined atmosphere, which could be far from the progressive and non-simultaneous release of trace amounts of VOCs from fresh plant material, both in vitro or into the soil [18].

### 2.2. Phytotoxicity of the Mimicked Phenolic Fraction

**Figure 2:**
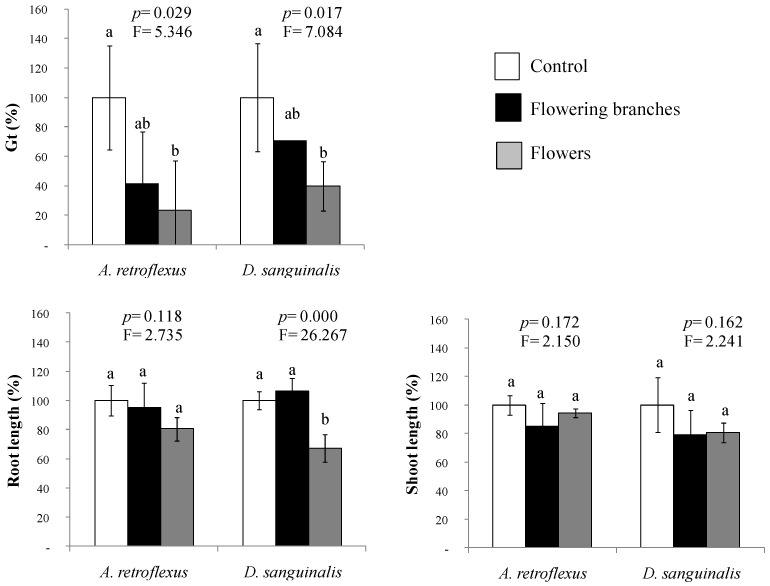
Effects of a mixture of phenolic compounds, prepared by mimicking the aqueous extract of *Cytisus scoparius* flowering foliage or only flowers, applied in Petri dishes on the total percentage of germinated seeds (Gt) and early growth of two agricultural weed species (*Amaranthus retroflexus* and *Digitaria sanguinalis*). Mean values of four replicates are represented in percentage relative to control. Error bars represent standard deviation (S.D.). For each target species, *p*- and F-values of the effects of treatments from one-way ANOVA or Kruskal-Wallis *H* tests are shown. Mean values labeled with distinct letters denote statistically significant differences among treatments (post hoc LSD or Tamhane’s *T*2 test).

The mimicked phenolic fraction probed in Petri dishes, where the mixtures of phenolic acids and flavonoids were prepared at the natural proportions and concentrations of *C. scoparius’* aqueous extracts, are represented in Figure 2. After the ANOVA, the germination of *A. retroflexus* and *D. sanguinalis* was significantly inhibited by 76% and 60% (*p* ≤ 0.05), respectively, by the mixture of phenolic compounds present in the *C. scoparius’* flower extract. In addition, root lengths of *D. sanguinalis* were also reduced by 33% relative to the control.

The results obtained did not entirely reproduce the effects of the aqueous extract of flowering branches and flowers described in Pardo-Muras et al. [23], at least for *A. retroflexus*. Here, only the mixture mimicking the aqueous extract of flowers was phytotoxic for germination of this species, showing no effect on growth. Such discrepancy suggests the participation of some other compounds with relevant phytotoxic or synergistic roles that were not detected or analysed from our aqueous extracts. Indeed, some phenolic compounds non-identified here, namely genistein or rutin, have been described in the literature for the aerial parts of *C. scoparius* [31]. Other phytotoxic allelochemicals belonging to other non-analysed chemical classes (e.g., terpenes, alkaloids) could also be present in the aqueous extracts; moreover, for *D. sanguinalis* growing in Petri dishes, the mimicked mixtures reproducing the *C. scoparius* aqueous extracts showed less statistically significant phytotoxic effects than the natural aqueous extracts studied in Pardo-Muras et al. [23] but similar in trend and magnitude. What does seem clear is that the phytotoxicity of both the phenolic mixtures and the natural extracts of *C. scoparius* may not be due to the action of a single phenolic compound but may be caused by the combined effect of several of them. For instance, the phenolic acids caffeic, *p*-coumaric, ferulic, trans-cinnamic, ellagic, and the flavonoids kaempferol and luteolin previously assayed alone at concentrations lower than 1 mM [23] did not show significant phytotoxicity on the germination and root growth. Since the concentrations of each of them are much lower in the aqueous extracts or their mimicked mixtures, the effects observed here should be most probably attributed to the interactions into the cocktail of phenolic compounds.

There is some evidence in the literature for increased inhibitory action in mixtures of phenols. For example, Blum [32] observed that as the number of phenolic acids (*p*-hydroxybenzoic, vanillic, ferulic, and *p*-coumaric acids) involved in a mixture increased the concentration of each compound in the mixture required to produce growth inhibition on *Cucumis sativus* decreased. Then, the author suggested that the phytotoxicity of a single compound is not predictive of the whole mixture bioactivity. Previously, Li et al. [33] demonstrated that the phenolic acids caffeic, ferulic, and cinnamic acids increased the effects of trans-cinnamic and abscisic acids on lettuce. In the same line, Chaves et al. [34] described higher levels of phytotoxicity of the phenolic acids ferulic, cinnamic, 4-hydroxybenzoic, hydroxycinnamic, oxalic, methylmalonic, *p*-anisic, and 3-hydroxybutyric when acting together on *Rumex crispus* germination and seedling growth than when they were assayed separately. Likewise, Harun et al. [35] described synergies among phloridzin and catechin, or *p*-coumaric and ferulic acids, identified from *Chrysanthemoides monilifera* subsp. *monilifera* by using their natural concentrations in the methanolic extracts of different plant parts. Conversely, Feitoza et al. [36] reported some antagonisms between *p*-coumaric acid and the flavonoids quercetin, luteolin, and kaempferol when assayed on the early growth of *Calopogonium mucunoides*.

### 2.3. Interactions between the Volatile and the Phenolic Mimicked Fractions

To the best of our knowledge, concerning the preparation and bioassay of mixtures of compounds, only Araniti et al. [29] and Harun et al. [35] considered their natural concentrations present in the plant extracts, the former using terpenoids dissolved in ethanol and then added to agar, and the latter using four phenolic compounds dissolved in distilled water; however, there are still no reports on the activity of certain VOCs in combination with water-soluble compounds, and of course, not in their natural proportions either; although, if complex mixtures mimicking natural plant profiles are recognized to be challenging to prepare *per se*, it would be even harder to reproduce their way of release (i.e., volatilisation and leakage from tissues) into their natural solvents in Nature (i.e., air and water, respectively). We used DMSO and a buffer [37] due to the lack of water solubility of some pure phenolic compounds. Also, the extremely low concentrations of VOCs potentially emitted by the flowering foliage are hard to achieve. In our approach, by applying 1 µL of the *C. scoparius* VOCs mixture inside the hermetic chambers, we had 0.23, 0.14, 0.34, and 0.29 ppm of verbenone, *α*-terpineol, linalool, and terpinen-4-ol, respectively, released at 1 L atmosphere; thus, despite the conservation of their natural proportions in the volatile extract, their experimental concentrations could exceed those released from the fresh plant material in the chamber over the 48 hours’ duration of the bioassay. 

**Figure 3:**
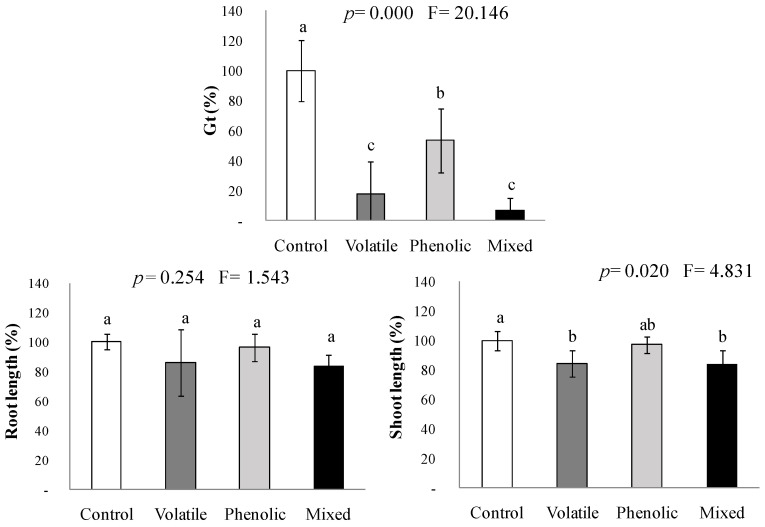
Effects of mimicked volatile and mimicked phenolic fractions of *Cytisus scoparius*, applied separately or concomitantly (mixed) in 1 L hermetic chambers, on the total percentage of germinated seeds (Gt) and early growth of *Amaranthus retroflexus*. Mean values of four replicates are represented in percentage relative to control. Error bars represent standard deviation (S.D.). *p*- and F-values of the effects of treatments from by one-way ANOVA or Kruskal-Wallis *H* tests are shown. Mean values labeled with distinct letters denote statistically significant differences among treatments (post hoc LSD or Tamhane’s *T*2 test).

Figure 3 shows the effects of the previously identified mimicked volatile and phenolic profiles in *C. scoparius* on the germination and early growth of *A. retroflexus*, acting either separately or concomitantly (volatile, phenolic, and mixed bioassays). Considering the experimental limitations discussed above, we found that the concentration of 1 ppm of the mimicked VOCs mixture inside the chamber inhibited *A. retroflexus* germination by 80% (*p* ≤ 0.001), but just 16% of the shoot elongation (*p* ≤ 0.05). The effects differed from those previously reported by Pardo-Muras et al. [22] for naturally emitted VOCs (no impact and 50% inhibition, respectively); thus, we do not discard other compounds in the natural volatile profile of *C. scoparius* interacted antagonistically or synergistically on different physiological processes, despite having been previously described as innocuous when applied alone [22]. 

The mimicked phenolic mixture in the hermetic chambers reproduced the trend observed when applied in Petri dishes, with a significant effect on *A. retroflexus* germination. This 46% inhibition of germination was similar to that reported in previous works for the natural aqueous extract [23]; however, contrary to the natural extract, the mimicked phenolic mixture was harmless to growth (Figure 3). Arroyo et al. [38] found similar results when they studied the autotoxicity of the aqueous extract of *Artemisia herba-alba* and suggested that pure compounds in the mimicked mixtures probably were degraded faster than in the natural aqueous extracts. Therefore, some natural synergistic interactions could have been absent in the mimicked phenolic mixture.

In the subsequent bioassay, when the mimicked VOCs mixture of *C. scoparius* was assayed in combination with the mimicked phenolic mixture, their joint action inhibited germination by up to 90% (*p* ≤ 0.001; Figure 3). Moreover, the few seeds of *A. retroflexus* that could have germinated under their combined effects would still suffer a 16% inhibition of shoot growth (*p* ≤ 0.05). No significant interactive effects were found between the volatile and the phenolic mixtures acting concomitantly on shoot growth; so, from this experiment, only the volatile compounds can be blamed for inhibiting the shoot growth. In contrast, the phytotoxicity on germination appeared to rely on the additive effects of volatiles and phenolics, with no evidence of synergistic interaction between both mimicked fractions. 

### 2.4. Interactions among Volatile and Water-Soluble Compounds Naturally Released from Cytisus Scoparius

**Figure 4:**
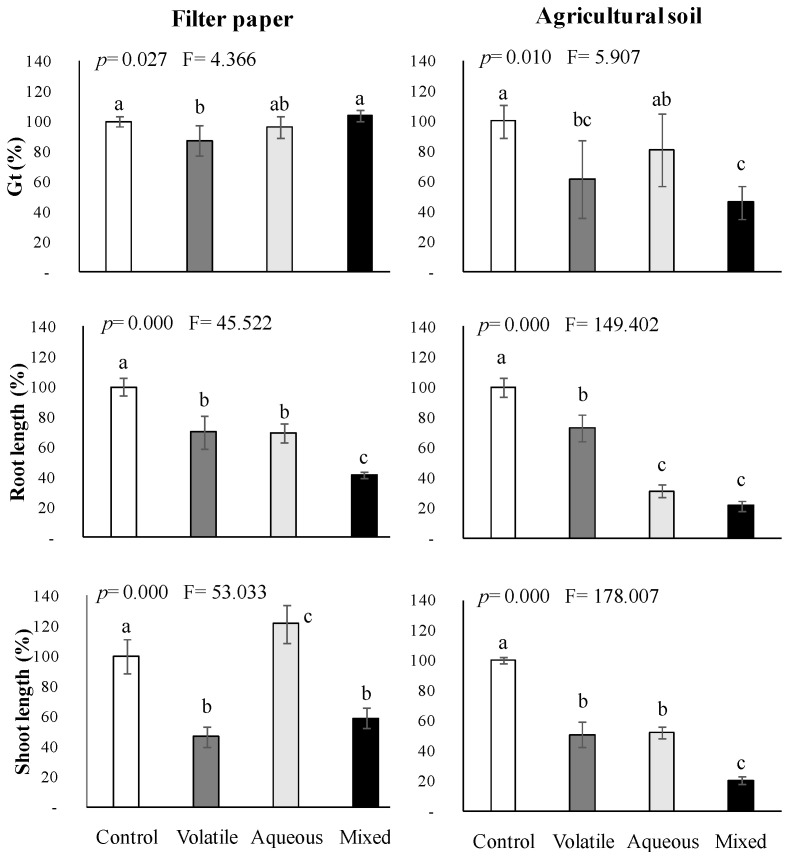
Effects of volatile compounds naturally released from the flowering foliage of *Cytisus scoparius* and its aqueous extract, acting separately or concomitantly in 1 L hermetic chambers, on the total percentage of germinated seeds (Gt) and early growth of *Amaranthus retroflexus* placed on filter paper or agricultural soil. Mean values of four replicates are represented in percentage relative to control. Error bars represent standard deviation (S.D.). *p*- and F-values of the effects of treatments from one-way ANOVA or Kruskal-Wallis *H* tests are shown. Mean values labeled with distinct letters denote statistically significant differences among treatments (post-hoc LSD or Tamhane’s *T*2 test).

In our conceptual framework, focusing on the use of the foliage of allelopathic species for field weed control, we followed the warnings of Inderjit et al. [6,10] on the need for realistic laboratory bioassays to elucidate the natural implications of allelopathy in agriculture. Hence, we undertook the calibration of all the results obtained from the mimicked mixtures against the natural volatile emission from fresh flowering foliage of *C. scoparius* and potentially leached water-soluble compounds, either acting separately or concomitantly (natural volatile, aqueous, and mixed bioassays) on the germination and early growth of *A. retroflexus*. Moreover, we also introduced the claimed soil factor [6]. These results are represented in Figure 4.

So far, only an approach by Dias and Moreira [15] studied the interactions between the volatile compounds emitted by a plant and its aqueous extract. These authors discovered that the volatiles emitted by *Cistus ladanifer* intact leaves inhibited the germination of *Trifolium subterraneum*, but only when the water-soluble compounds were present. From our results (Figure 4), when assayed on filter paper, we found significant phytotoxic interactions between the naturally emitted VOCs and the aqueous extract on the root elongation of *A. retroflexus*, attaining 60% inhibition (*p* ≤ 0.001). Conversely, inhibition of germination and shoot growth of the seeds placed on filter paper was only attributed to the VOCs released into the hermetic chamber; however, when added to fresh soil, and according to Pardo-Muras et al. [23], the aqueous extract of *C. scoparius* became significantly more effective in preventing root and shoot elongations. This time, excitingly, the soil appeared to promote synergistic interactions among the released volatiles and the water-soluble compounds, together being capable of inhibiting the root and shoot growths of *A. retroflexus* by 80% (*p* ≤ 0.001) and preventing germination by more than 50% (*p* ≤ 0.01), despite the shallow natural concentration of each compound in the natural cocktail.

Chemical ecologists have warned about the extrapolation of lab results to Nature since allelochemical compounds tested in vitro usually have weak or no phytotoxicity in the field [6,16,39,40] because of degradation by microorganisms and/or adsorption to soil particles. Some authors have addressed the fate of some water-soluble and volatile allelopathic compounds once released into the soil [14,18] but, as far as we know, no previous study was performed on their phytotoxic interactions and drift into the soil. Thanks to the mimicked experiments, we can state that such interactions can exist among certain terpenes and phenolic compounds. Here, we contributed that soil can enhance the phytotoxicity of the complex cocktail of allelochemicals released from a plant, probably by fostering their multiple synergistic interactions and/or promoting some new ones (e.g., by physico-chemical activation and/or microbial metabolization [2,16]). Their full bioactivity appeared to be achieved only when the compounds interacted naturally into the soil matrix but not into artificial layers (e.g., filter paper). The magnitude and duration of the bioherbicide effects previously reported in pot experiments or at field scale [18,19,20,25] are consistent with current results.

## 3. Conclusions

The findings of this work confirm that the effectiveness of *Cytisus scoparius* foliage for weed control, and probably that of other allelopathic species, relies on complex, multiple synergistic interactions among volatile and water-soluble allelochemicals favored by the soil, thus giving us an idea of how complex the multiple and multi-scale interactions among phytochemicals and their fate in Nature could be. Although valuable and suitable for studying joint interactions, the approach of artificially mimicking the chemical composition of the aqueous extract or the VOCs profile of a given plant is still insufficient to display the whole phytotoxicity of the compounds acting together when released from the plant material. Several aspects require more innovative experimental approaches, such as VOCs’ and water-soluble phytochemicals’ physico-chemical and physiological transformations into the soil matrix [41]. Furthermore, reference models are necessary to understand the natural allelopathic relationships between plants and their abiotic and biotic environments in different ecosystems and agroecosystems. Of course, for the potential application of these powerful synergies for integrated weed management (i.e., crop rotation, green manuring, use of plant residues, or intercropping), including the search for new multi-mode-of-action herbicides with reduced risk of resistance development. Finally, the exploitation of the synergistic interactions among natural compounds of plant origin could be helpful in other frameworks that pursue new bioactive formulations with minimal concentrations of different active principles.

## 4. Materials and Methods

### 4.1. Standard Compounds, Plant and Soil Materials, and Target Species

All the compounds tested were previously identified from the volatile and aqueous extract of flowering branches of *C. scoparius*, obtained by GC-GC/MS and HPLC-DAD, respectively [22,23]. The pure volatile and phenolic compounds were commercially available and purchased from Sigma-Aldrich (St. Louis, MO, USA), except for the flavonoid ellagitannin, which was isolated from the aqueous extract of *Punica granatum* L. bark at Laboratorios Admira (Murcia, Spain). All the compounds were used as received without further purification.

Two agricultural weed species, *A. retroflexus* (redroot pigweed) and *D. sanguinalis* (large crabgrass) from Herbiseed (Twyford, England, UK), were used as representative dicot and monocot target species, respectively. For bioassays optimization, *A. retroflexus* seeds were pretreated for synchronization by soaking in distilled water for 15 days at 4 °C and then air-dried, whereas *D. sanguinalis* seeds were placed under light for 56 days at 4 °C.

The soil was collected from a superficial horizon in a cultivated field (42°12′15.6″ N 8°46′19.8″ W) dedicated to horticulture for fifteen years and then left fallow over the last two years. The soil was sieved through 2 mm mesh to remove debris and plant tissues. Soil physicochemical characteristics were pH (1:2.5 H_2_O) 6.2, EC < 0.16, organic matter 5.6%, total N 0.3%; concentrations of Ca^2+^, K^+^, Mg^2+^, Na^+^ 7.9, 0.8, 1.22, 0.14 cmol(+)·kg^−1^, respectively, and P^3−^ 82 mg·kg^−1^; and a maximum water retention capacity (WRC) of 340 mL·kg^−1^ dw.

Flowering branches (flowers, leaves, and shoots) of *C. scoparius* were collected during May 2019 in the vicinity of the University of Vigo (Galicia, NW Spain, 42°09′56.0″ N 8°41′04.7″ W). A sample of *C. scoparius* (n. 75594) was deposited in the herbarium (SANT) of the University of Santiago de Compostela (USC, Santiago de Compostela, Spain). Prior to bioassays, sub-samples of fresh flowering branches were weighed and then dried at 60 °C to constant weight to obtain the dry weight/fresh weight ratios.

### 4.2. Probing the Phytotoxicity of the Mimicked Volatile Fraction

Based on Pardo-Muras et al. [22], a mixture of VOCs with proven individual phytotoxicity was prepared by maintaining the natural proportions found in the volatile extract of *C. scoparius* flowering branches. The VOCs mixture was composed of verbenone, *α*-terpineol, linalool, and terpinen-4-ol (23:34:29:14), and the effects were evaluated on the germination and root and shoot lengths of *A. retroflexus* and *D. sanguinalis*. Seeds and seedlings of the target weed species were exposed to 1 or 2 µL of the volatile mixture (12.5 or 25 ppm in the Petri dish atmosphere, respectively [42]).

Twenty-five seeds were incubated in each Petri dish (9 cm diameter) for the germination bioassays, placed on a filter paper moistened with 4 mL of distilled water. A filter paper strip was fixed inside the plate’s top lid, and the corresponding concentration of the mixture was added to it with a micropipette. In such a way, seeds were exposed to the volatilised compounds inside the dish volume [22,24,43]. The control treatment consisted of Petri dishes without any added compound. Petri dishes were immediately closed and sealed with Parafilm and incubated in the dark at a constant temperature of 27 °C. The number of germinated seeds (rupture of seed coats and the emergence of radicle ≥1 mm; [44]) was counted every 12 h for *A. retroflexus* and every 24 h for *D. sanguinalis* until no more seeds germinated in the control dishes. The total percentage of germinated seeds (Gt) and the coefficient of the rate of germination (CRG) were calculated according to Chiapusio et al. [45] and De Bertoldi et al. [46], respectively.

For early growth bioassays, fifteen pre-germinated seeds (radicle length between 1 and 3 mm) were used under the same conditions as for germination bioassays. Root and shoot lengths of the pre-germinated seeds were measured after 48 h, and mean values per dish were expressed as a percentage of the respective control. Four replicates were carried out for each mixture, concentration, and target species.

### 4.3. Probing the Phytotoxicity of the Mimicked Phenolic Fraction

Based on the chemical profiles obtained from the aqueous extract of flowering branches or flowers of *C. scoparius* (at a plant dry weight/ distilled water ratio of 66.7 g·L^−1^; [23]), the following mixtures of phenolic compounds were prepared to mimic their natural concentrations: (i) flowering branches mixture, consisting of caffeic acid at 0.67 μg·mL^−1^, trans-cinnamic acid at 0.19 μg·mL^−1^, vanillin at 0.53 μg·mL^−1^, *p*-coumaric acid at 1.96 μg·mL^−1^, apigenin at 0.86 μg·mL^−1^, luteolin at 3.56 μg·mL^−1^, ellagic acid at 0.91 μg·mL^−1^, ellagitannin at 1.17 μg·mL^−1^, prunetin at 0.84 μg·mL^−1^, and naringenin at 0.07 μg·mL^−1^; and (ii) flowers mixture, consisting of caffeic acid at 2.86 μg·mL^−1^, trans-cinnamic acid at 0.68 μg·mL^−1^, vanillin at 0.18 μg·mL^−1^, *p*-coumaric acid at 6.11 μg·mL^−1^, ferulic acid at 0.49 μg·mL^−1^, luteolin at 28.83 μg·mL^−1^, ellagic acid at 2.09 μg·mL^−1^, ellagitannin at 4.05 μg·mL^−1^, prunetin at 0.99 μg·mL^−1^, and quercetin at 1.49 μg·mL^−1^.

The mixtures were prepared at controlled pH in a buffer 10^−2^ M 2-[N-morpholino] ethanesulfonic acid (MES) and 1 M NaOH (pH 6.0). Each pure compound was first solubilised in DMSO [37], and the corresponding stock solution was prepared at 2 mM in the buffer. Aliquots of the stock solutions were mixed and then diluted with the buffer to attain the actual concentration of each compound in the natural aqueous extract, with a final concentration in the buffer of 5 µL DMSO·mL^−1^. For each agricultural weed species, control treatments consisted of buffer at the same DMSO concentration.

For the germination and early growth bioassays, seeds and seedlings of the target weed species *A. retroflexus* and *D. sanguinalis* were incubated in 6-well plates placed on a layer of filter paper wetted with 750 μL of each mixture [42]. These bioassays were conducted and monitored as described in sub-Section 4.2. For each treatment and target species, four replicates were carried out.

### 4.4. Interaction Bioassays between the Volatile and the Phenolic Mimicked Fractions

One-liter hermetic chambers were used to dilute the *C. scoparius* VOCs mixture close to the natural concentrations of VOCs potentially emitted by the plant material. The germination and early growth bioassays were conducted with *A. retroflexus* due to its faster germination and growth rate. The following treatments were applied: (i) *Volatile bioassay*, in which seeds or pre-germinated seedlings were incubated on a filter paper layer wetted with 4 mL of the buffer solution used for preparing the phenolic mixtures (5 µL DMSO solution mL^−1^ at pH 6); then, 1 µL of the *C. scoparius* VOCs mixture [i.e., verbenone: *α*-terpineol: linalool: terpinen-4-ol (23:34:29:14)] was added with a micropipette to a filter paper strip hanging from the cover inside the hermetic chamber, which was immediately closed. In this way, the VOCs were left to release at a maximum total concentration of 1 ppm into the chamber atmosphere (i.e., 1 µL·L^−1^). (ii) *Phenolic bioassay*, in which seeds or pre-germinated seedlings were incubated on a filter paper layer wetted with 4 mL of the mixture of phenolic compounds that mimicked the phenolic profile of *C. scoparius* flowering branches, prepared as described in Section 4.3. An untreated filter paper strip was hung from the cover inside the hermetic chamber. And (iii) *Mixed bioassay*, in which seeds or pre-germinated seedlings were incubated on a filter paper layer wetted with 4 mL of the mixture of phenolic compounds of *C. scoparius* flowering branches, besides a filter paper strip with 1 µL of the *C. scoparius* VOCs mixture hung from the cover. The control experiment consisted of seeds or pre-germinated seedlings incubated on a filter paper layer wetted with 4 mL of the buffer solution (5 µL DMSO solution mL^−1^ at pH 6) and a hanging untreated filter paper strip.

Seeds and seedlings were incubated and monitored as described for the previous bioassays. Each treatment was replicated four times.

### 4.5. Interaction Bioassays among Volatile and Water-Soluble Compounds Naturally Released from Cytisus Scoparius

The whole potential natural interactions among the volatile compounds emitted by fresh flowering foliage of *C. scoparius* and its water-soluble compounds were experimentally reproduced to calibrate the responses obtained from the mimicked volatile, phenolic, and mixed bioassays. For the aqueous extract preparation, the fresh flowering branches were soaked in distilled water at a plant dry weight/distilled water volume ratio of 66.7 g·L^−1^ [23] in the dark at room temperature for 24 h and filtered through a 0.45 μm cellulose membrane. A battery of bioassays was performed under the same conditions described for Section 4.4 on the target species *A. retroflexus*. The following treatments were applied on filter paper or fresh agricultural soil: (i) *Natural volatile bioassay*, in which seeds or pre-germinated seedlings were incubated on the substrate (filter paper or soil) wetted with distilled water. Then, fresh flowering branches equivalent to 2 g of dry weight [i.e., 7.2 g fw] were wrapped in a sterile cotton gauze swab and hung from the cover inside the hermetic chamber, which was immediately closed. (ii) *Natural aqueous bioassay*, in which seeds or pre-germinated seedlings were incubated on the substrate wetted with the aqueous extract. A cotton gauze swab containing drinking straws cut into pieces at the same volume as fresh plant material was hung from the cover inside the hermetic chamber. And (iii) *Natural mixed bioassay*, in which seeds or pre-germinated seedlings were incubated on the substrate wetted with the aqueous extract of *C. scoparius* flowering branches, besides fresh plant material of *C. scoparius* equivalent to 2 g dw hung from the cover. The control treatment consisted of seeds or pre-germinated seedlings incubated on filter paper or soil layer wetted with distilled water and a hanging cotton gauze swab containing pieces of drinking straws. For each bioassay, hermetic chambers were fitted with ten filter paper layers or 50 g of agricultural soil, both substrates with 22% moisture content, wetted with 18 mL of the aqueous extract or distilled water, as appropriate. In such a way, the seeds were exposed to enough solution quantity to allow imbibition and to the same final extract concentration (41.4 g dw·L^−1^).

Four replicates were carried out for each treatment and substrate (filter paper or soil).

### 4.6. Statistical Analysis

Replicated experiments were conducted in a completely randomised design. Data were expressed as a percentage relative to control. After testing for normality by Kolmogorov-Smirnov test and for homogeneity of variance by Levene’s test, data were analysed by one-way ANOVA (*p* ≤ 0.05) and LSD test (*p* = 0.05) for post hoc multiple comparisons. In the case of heteroscedasticity, the variance was analysed by Kruskal-Wallis *H* test and Tamhane’s *T2* for post hoc multiple comparisons.

Statistical analyses were carried out using the SPSS v.19 software (IBM SPSS Inc., Chicago, IL, USA) for Windows.

## Figures and Tables

**Table 1:** Effects of a mixture of VOCs, prepared by mimicking the volatile profile of *Cotises scoparius* flowering foliage or only flowers, assayed at 12.5 and 25 ppm in Petri dishes, on the germination index (CRG) of two agricultural weed species (*Amaranthus retroflexus* and *Digitaria sanguinalis*). Values represent the means of four replicates ± standard deviation (S.D.). For each species, mean values labeled with distinct letters are significantly different at *p* ≤ 0.05 (post hoc LSD or Tamhane’s *T*2 test).

	*Amaranthus retroflexus* CRG	*Digitaria sanguinalis* CRG
Control	2.76 ± 0.01 a	1.36 ± 0.02 a
12.5 ppm (1 µL)	0.59 ± 1.19 b	1.21 ± 0.08 b
25 ppm (2 µL)	0.64 ± 1.28 b	1.17 ± 0.07 b
